# Promoting children’s health through community-led street interventions: analyzing sustained voluntarism in Canadian School Streets

**DOI:** 10.1186/s12889-024-18531-9

**Published:** 2024-04-11

**Authors:** Carise M. Thompson, Patricia A. Collins, Katherine L. Frohlich

**Affiliations:** 1 Department of Geography and Planning, Queen’s University, Kingston, Ontario Canada; 2 Centre de recherche en santé publique (CReSP), Université de Montréal, Montréal, Québec Canada; 3 École de Santé PubliqueUniversité de Montréal, Montréal, Québec Canada

**Keywords:** Community health promotion, Volunteers, Active transport, Ottawa Charter, Healthy communities, School Streets

## Abstract

**Background:**

Active School Travel (AST) initiatives align with the Ottawa Charter for Health Promotion, which calls for ‘creating supportive environments’ and ‘strengthening community action.’ However, their reliance on volunteers poses sustainability challenges. The main objectives of this study were to document the motivations, satisfaction, and experiences of volunteers involved in sustaining two AST initiatives in Ontario for an entire school year.

**Methods:**

Two volunteer-led School Street initiatives in Kingston, Ontario successfully operated during pick-up and drop-off times of each school day. The first initiative operated for the entire 2021-2022 school year, and the second operated for the entire 2022-2023 school year. These initiatives were the first of their kind in the province of Ontario, Canada. Volunteers from both sites (*n* = 56) participated in online surveys and their motivations, satisfaction, and experiences of their role were compared using the 2-sided Fisher’s Exact Test.

**Results:**

Over 80% of volunteers were highly motivated to promote safety and over 70% of volunteers were highly motivated to disrupt the status quo of unsupportive, car-centric urban environments by reimagining how streets can be used. By taking collective action to re-shape the environment around these public schools to support healthy, active living, our findings reveal that over 90% of volunteers were highly satisfied. Of the volunteers, 87% felt they contributed to child safety and 85% felt they had developed stronger community connections. They appreciated the short (i.e., 40 minute) time commitment of each shift, weekly email communications by the community organization leading the initiative, and the volunteer schedule. They also appreciated the positive social interactions during volunteer shifts, which they felt outweighed the minimal resistance they experienced.

**Conclusions:**

This research demonstrates the importance of logistical, motivational, and social factors in recruiting and retaining volunteers for community-led School Streets. Our findings support appealing to prospective volunteers’ influence in achieving School Street objectives (e.g., improved safety) in recruitment efforts, as well as highlighting School Streets’ innovative approach. Communicating with volunteers throughout School Street planning and implementation processes and limiting traffic in the closed street zone (i.e., by excluding the school staff parking lot and private driveways from the scope) are additional recommendations based on the findings of this study.

**Supplementary Information:**

The online version contains supplementary material available at 10.1186/s12889-024-18531-9.

## Background

The 1986 Ottawa Charter for Health Promotion advocated for action on and across five pillars to achieve health for all: building healthy public policy, creating supportive environments, strengthening community actions, developing personal skills, and reorienting health services [1, 2]. These action areas emphasize a societal approach to health promotion whereby health is envisioned as a shared responsibility. While building healthy public policy and reorienting health services focus action at system levels, the pillars of creating supportive environments and strengthening community action urge communities, and the citizens within them, to assert control over their own health and well-being [2].

According to Fry and Zask’s [3] analysis of these action areas, creating supportive environments is defined as *“developing physical and/or social environments in ways which support health and protect against physical hazards and socially and psychologically damaging practices”*. Creating, modifying, or expanding upon existing infrastructure, programs, and services are examples of how this can be done [3]. Strengthening community actions refers to *“expanding the resources and capacity of communities to make decisions and take collective action to increase their control over the determinants of their health”* [3]. For example, communities – people who share a sense of identity which may or may not be tied to geographic locality – may work together to develop programs or networks, advocate for service or program improvements, or for organizational or public policy change [3].

Active School Travel (AST) initiatives are child health promotion interventions that encompass both ‘strengthening community action’ and ‘creating supportive environments’. These initiatives aim to increase children’s engagement in AST by making walking, cycling, wheeling, and rolling to school safer and more desirable [4]. AST initiatives are diverse, spanning group walking and bicycling programs, wayfinding, crossing guards, and park and strides, with each approach emphasizing different determinants of AST (e.g., encouragement, engineering, enforcement, education) [5, 6]. Yet despite this diversity, citizen volunteers are often the backbone of AST initiatives. Accordingly, promoting AST manifests through the collective action of ordinary citizens, such as parents and residents, to create child-friendly environments that support children’s health and well-being. AST initiatives have also been shown to foster a sense of social connection within neighbourhoods [7, 8], which can further enhance community participation and civic engagement.

Given their reliance on citizen volunteers, attracting and retaining volunteer support over the long-term is the primary challenge to sustaining AST initiatives [9, 10]. Initiatives like the walking school buses and park and strides require a substantial number of volunteers to successfully reduce barriers to active travel (e.g., by providing adult supervision on the trip to school). This limitation is not unique to AST initiatives, and the broader literature on volunteerism suggests that motives for volunteering, competing priorities, and satisfaction contribute to volunteer retention issues [11–13].

Encouraging and maintaining social connection among volunteers and the community may attenuate this relationship. Research has shown that social connectedness is a strong predictor for volunteer satisfaction [14], duration of volunteer commitment [15] and lower burnout among volunteers [16]. Moreover, it has been established that social connection is an important factor in fostering collective action, especially for informal, grassroots-led organizing [17, 18]. Social connection in community health promotion programs has been positively associated with local community participation, leadership, volunteerism, and reciprocity, for example [19].

Another potential solution to volunteer retention issues is increasing volunteer engagement through work that is socially innovative. According to Westley & Antadze (2010), a social innovation is any initiative (product, process, program, project, or platform) that challenges and, over time, contributes to changing the defining and authority flows or beliefs of the broader social system in which it is introduced [20]. Other research suggests that social innovation may stimulate intrinsic motivation among volunteers by promoting autonomy, competence and relatedness [21], and may lead to broader societal benefits, such as community resilience [22]. A growing number of Canadian and European communities are pilot-testing an innovative approach to promote AST called ‘School Streets’ [23].

School Streets are initiatives that create a car-free environment in front of schools at the start and end of the school day to enable children and their caregivers to come and go from school safely and actively [24]. School Streets are growing in popularity in response to high levels of vehicular congestion and dangerous maneuvers by motorists around elementary schools, coupled with declining rates of AST [23, 25, 26]. In 2019 and 2020, the cities of Toronto, Victoria, and Vancouver implemented short-term School Street initiatives, ranging in duration from one day to one month [23], while Canada’s first year-long School Street initiative was implemented in Winnipeg during the 2020-2021 school year [27]. The success of these preliminary School Street pilots has led to the implementation of additional School Streets in these and other cities in Canada [23]. As innovative AST initiatives like these continue to grow in popularity, research is needed to better understand the experiences of volunteers who contribute to longer-term AST initiatives that seek to promote children’s health and well-being.

In the mid-sized Canadian city of Kingston, Ontario, two volunteer-led School Streets, School Street A and School Street B, operated for the duration of the 2021-2022 and 2022-2023 school years, respectively. Both School Streets were piloted in these communities as part of a larger population health intervention research project called *Levelling the Playing Fields*, which seeks to evaluate the implementation and outcomes associated with street closure interventions that aim to increase children’s engagement in active travel, independent mobility, and free play. Through this project, School Street A became the first to run for an entire school year in the province of Ontario. Subsequently, School Street B was implemented at a different school in the same city and ran for an entire school year. Community volunteers played a crucial role in both School Street A and B’s year-long operation by dedicating themselves to running the initiative. An assets-based approach is applied here to understand the factors that contributed to the successful, sustained operation of these two School Streets. Specifically, we surveyed volunteers of both of Kingston’s School Streets to learn about their: (i) motivations for volunteering for the School Street; (ii) satisfaction with the volunteer position; (iii) experiences while volunteering for the School Street; (iv) reasons for discontinuing their involvement as a volunteer (if applicable); and (v) recommendations for improving the volunteer experience. The findings of this research offer critical insights into the role of volunteers in sustaining an innovative AST intervention, and in contributing to the goals of the Ottawa Charter for Health Promotion.

## Materials and methods

### School street intervention context

**Fig. 1:**
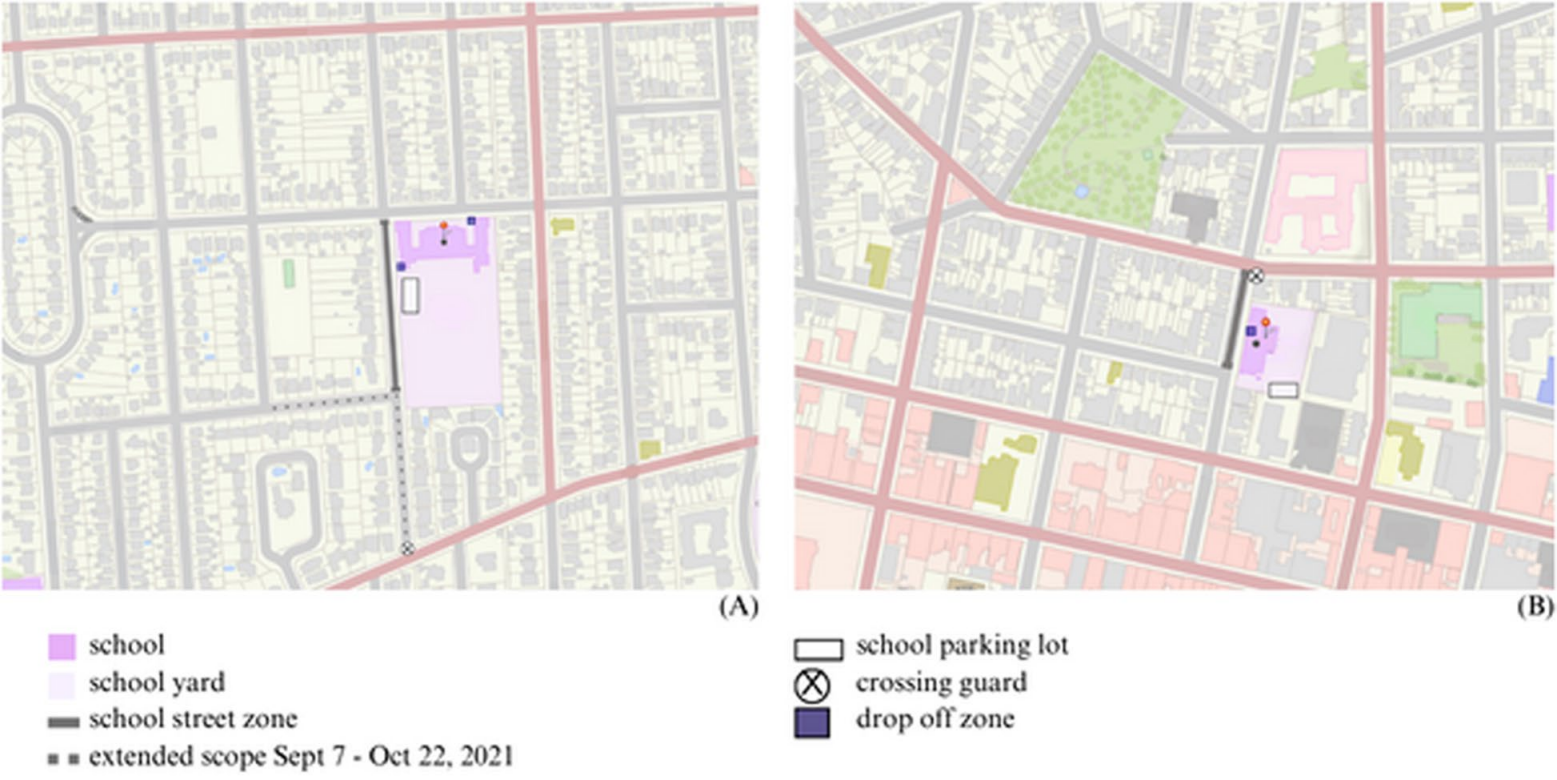
Topographic maps of School Street (**A**); and School Street (**B**) which operated for the duration of the 2021-2022 and 2022-2023 school years, respectively

The School Street A pilot operated during the 2021-2022 school year at a school located within 2 kilometers of the city’s central business district and a university (Fig. 1). The intervention involved closing a 192-metre portion^1^ of the street adjacent to the selected elementary school, encompassing the main drop-off and pick-up zone for students as well as the staff parking lot. The closed zone did not include the kindergarten drop off zone located on the North side of the school (Fig. 1). The School Street B pilot operated during 2022-2023 at a school located in the heart of the city. The intervention involved closing a 94-metre stretch of street adjacent to the school which encompassed the school’s only drop-off and pick-up zone. In contrast to School Street A, school staff at the School Street B site did not have to pass through the zone to enter and exit the staff parking lot.

For each intervention, the School Street was overseen by a community-based active transportation organization that bore responsibility for procuring the street closure permit and equipment, assuming liability, and managing the volunteer schedule. All volunteers met minimum age requirements (i.e., 18 years of age or older), completed a valid background check through the local police department, and completed volunteer traffic control training. Volunteers were not paid, but fees associated with their background checks were compensated. To minimize and calm vehicular traffic within the zone, only exempted motorists (e.g., school staff, residents within the closed road) were permitted to enter and exit the School Street zones during the intervention operations, and were required to travel at a walking pace, alongside a volunteer. In addition to chaperoning exempt vehicles through the zone, volunteer responsibilities at each intervention site consisted of gathering, transporting, and setting up equipment; safely directing and regulating traffic flow away from the School Street; answering questions about the initiative; and taking down and storing equipment. The School Street operation times and days, volunteer shift length, and equipment used were identical in each intervention, however the smaller scope of School Street B meant that the number of volunteers required for each shift was slightly less than the number required for School Street A (i.e., 2 versus 3 volunteers per SS shift). Additional details regarding the School Street sites are provided in Table 1.

**Table 1:** Characteristics of School Streets, School Profiles, and Surrounding Neighbourhoods by School Street Site

		SS Site A	SS Site B
SS Characteristics	Span of SS intervention	192 metres	94 metres
	SS operation times	0840-0910, 1520-1550	
	Volunteer shift length	40 minutes	
	SS operation days	Monday - Friday	
	Year of operation	September 2021 - June 2022	September 2022 - June 2023
	Volunteer requirements	3 per SS shift	2 per SS shift
	Volunteer responsibilities	equipment set-up; chaperone exempted motorists through SS zone at walking pace and alert pedestrians in SS to move to sidewalk for motorists to pass; direct and regulate traffic flow away from the SS; answer community members' questions about the SS; equipment take-down	
	Equipment	plastic A-frame barricades, road closed signs, portable sign stand	
	Equipment storage locations	school property	
	Programming (e.g., games, activities)	no coordinated programming	
School Characteristics	School size (23/24)	232	~250
	Grades (age range)	K-6 (4-12 years)	K-8 (4-14 years)
	Bussing eligibility	<5% of students	
	School day schedule	0900-1530	
Surrounding Neighbourhood Characteristics [28]	Population density	2,035/km^2^	4,695/km^2^
	Single family dwellings	74%	9%
	Average household size	2.5	1.7
	Median household income in school's census tract	$121,000	$54,800
	Home ownership rate	74%	29%
	Labour participation rate	61%	69%
	Commute by automobile	51%	41%

*SS* School Street

High concentration of children living in the neighbourhood, high levels of traffic congestion adjacent to the school at drop-off and pick-up times, ease of traffic rerouting, and buy-in from the school principal were all necessary requirements for selecting intervention sites. Additionally, the research team was keen to evaluate the implementation and outcomes of School Streets in socioeconomically contrasting neighbourhoods. As such, School Street A operated in a neighbourhood where the median household income in 2020 was $121,000, while School Street B operated in a neighbourhood where the median household income was $54,800 [28]. Table 1 also contrasts the census tracts of each school site in terms of population density, percentage of single family dwellings, and home ownership rates [28].

### Participant recruitment

School Street A involved 32 volunteers over the course of the school year, and School Street B involved 25 volunteers. All volunteers from both sites were invited to participate in an online survey to share their perspectives, and 100% of the volunteers completed the survey. Email invitations were distributed to the community organization’s listserv of School Street volunteers by P.C. including an anonymous link to the survey, and a reminder email was later sent by the volunteer coordinator. Ethics approval for this study was obtained from the Queen’s University General Research Ethics Board.

### Data collection

Identical surveys were distributed to volunteers from School Streets A and B between May and June 2022 and 2023, respectively. Surveys were administered online and completed anonymously using Qualtrics software. Volunteers were asked a mix of multiple choice, Likert scale, and open-ended questions. All survey questions were developed by the first and second authors and were designed to assess the specific motivations for becoming a School Street volunteer, satisfaction with the experience as a volunteer, frequency of issues encountered during volunteer shifts, reasons for discontinuing their involvement as a volunteer (if applicable), and ideas for improving the volunteer experience (Additional file 1: Appendix A). Development of survey questions drew from established methods in survey design, as described by Dillman et al. [29] and Fowler [30]. Existing validated scales were not used because of the high level of specificity required in the survey questions about the School Street intervention, and because the goal of the survey was not to make inferences about a broader population of volunteers. The instrument was subjected to peer review within the *Levelling the Playing Fields* team, but it was not pilot tested among the already small study population in order to maximize the total number of completed surveys that the research team could collect.

*Volunteer motivation* was assessed on 17 items focusing on the objectives of the School Street, logistical factors associated with the intervention, and opportunities the volunteer experience afforded. *Satisfaction* was assessed on 19 items pertaining to overall satisfaction, satisfaction with specific aspects of the position, and agreement with outcomes related to volunteer work. *Issues encountered* was measured with 7 items focused on frequency of encountering difficult situations during volunteer shifts and *volunteer dropout factors* was measured with 10 items regarding individual-level factors and aspects associated with the volunteer position. *Ideas for improvement* was measured by 3 items related to recognition and sustainability. Five-point scales were used for questions on which greater variation in responses was expected (i.e., *Satisfaction*, *Issues Encountered*), otherwise three-point scales were used to minimize respondent burden as much as possible. Survey respondents were asked to elaborate on closed-ended questions through seven open-ended questions. Given how familiar the research team was with many of the volunteers, the survey was administered anonymously, so that volunteers could provide as honest an assessment as possible. Specifically, the survey did not capture email addresses, gender, age, or any other demographic information. There was no incentive for participating in the survey. To prevent multiple submissions, a security feature was enabled within the survey software.

### Data analysis

Survey responses from School Street A and B volunteers were compared for the questions about motivations, satisfaction, experience, and dropout using the 2-sided Fisher’s Exact Test, and p-values less than 0.05 were considered significant. Statistical analyses were performed using IBM SPSS version 29. All scales were dichotomized as follows: *motivations* into the categories of “very influential” (previously strongly influential) and “not very influential” (previously modestly influential and not influential); *satisfaction* into the categories of “very satisfied” (previously extremely satisfied and very satisfied) and “not very satisfied” (previously somewhat satisfied, not very satisfied, and not at all satisfied); *issues experienced* into the categories of “never/rarely” and “frequently” (previously sometimes, often, and always); and *volunteer dropout factors* into the categories of “very influential” (previously strongly influential) and “not very influential” (previously modestly influential and not influential). Perceived outcomes were recoded into the two categories “agree” (previously strongly agree and agree) and “disagree/neutral” (previously strongly disagree and disagree). Open-ended responses were reviewed and supplemented the quantitative survey results to add depth to our understanding of volunteers’ motivations for joining the initiative, satisfaction, experiences associated with serving as a School Street volunteer, and reasons for dropout.

## Results

The study results are presented in the following six sections: 1) participant characteristics, 2) motivations, 3) satisfaction, 4) experiences, 5) dropout factors, and 6) considerations for future initiatives. In each section, survey trends are first reported, followed by complementary quotations from the open-ended survey responses. For most items on the survey, there were no significant differences in responses between volunteers from each intervention site; items on which there were significant differences between sites are discussed.

### Research participants

In total, 56 volunteers from 2 School Street sites participated in the anonymous online survey, 31 (55%) of whom volunteered at the School Street A site and 25 (45%) of whom volunteered at the School Street B site. While demographic questions were omitted, survey participants were asked generally about how they identify themselves in relation to the intervention. Volunteers were diverse, identifying themselves as parents or grandparents of students (59%), residents of the area (36%), university students or alumni (23%), and AST advocates (21%). Most participants took on one (59%) or two shifts (29%) per week, and over 69% of participants volunteered for 7 months or longer.

### Volunteers’ motivations

The motivations of volunteers are provided in Table 2. Across School Street sites, promoting safety around schools was the strongest motive for volunteering (84% very influential), followed by re-imagining how streets can be used (71% very influential). Volunteers from School Street A were more strongly motivated by the objectives to promote AST (*p*=0.015) and physical activity among children (*p*=0.007). On the other hand, School Street B volunteers were more strongly motivated by the opportunity to give back to their child(ren)’s school (*p*<0.001) and their community (*p*=0.031). Of the logistical and other factors, shift length, proximity to home, and ease of work involved were the most influential motivating factors across both groups of volunteers.

**Table 2:** Statistical Comparisons of Volunteers’ Motivations (*n* (%) reporting ‘very influential’) by School Street Site

Survey Question	Category	Pooled Total (*n*=56)	SS A (*n*=31)	SS B (*n*=25)	Chi-Square Statistic	Fisher's Exact Test (2-sided) *p*-value
		*n* (%)	*n* (%)	*n* (%)		
How influential were the following School Street program objectives in motivating you to volunteer for this program?	Promoting safety around schools	47 (84)	25 (81)	22 (88)	0.555	0.716
	Re-imagining how streets can be used	40 (71)	23 (74)	17 (68)	0.26	0.767
	Promoting active school travel	31 (55)	22 (71)	9 (36)	6.847	**0.015**
	Promoting children’s independent mobility	25 (45)	15 (48)	10 (40)	0.394	0.596
	Promoting physical activity among children	25 (45)	19 (61)	6 (24)	7.787	**0.007**
How influential were these other factors in motivating you to volunteer for this program?	Length of volunteer shifts	31 (55)	18 (58)	13 (52)	0.206	0.788
	Proximity of the School Street to my home	28 (50)	17 (55)	11 (44)	0.65	0.591
	Opportunity to give back to my community	27 (49)	11 (36)	16 (67)	5.263	**0.031**
	Ease of work involved in a volunteer shift	23 (42)	13 (42)	10 (42)	0.000	1.000
	Frequency of volunteer shifts	23 (41)	12 (39)	11 (44)	0.16	0.787
	Opportunity to be involved in a novel program	22 (40)	15 (50)	7 (28)	2.75	0.166
	Opportunity to give back to my child(ren)’s school	22 (39)	6 (19)	16 (64)	11.565	**<0.001**
	Opportunity to be outdoors	19 (34)	11 (36)	8 (32)	0.075	1.000
	Opportunity to meet new people and socialize	14 (25)	7 (23)	7 (28)	0.217	0.759
	Opportunity to be involved with community organization	9 (16)	7 (23)	2 (8)	2.181	0.167
	Opportunity to gain volunteer experience for my career development	5 (9)	4 (13)	1 (4)	1.249	0.373

The *p*-values represent differences between volunteers of School Street A and School Street B; bolded *p*-values are statistically significant at *p*<0.05

*SS* School Street

In their open-ended responses, participants elaborated on why they were motivated to volunteer, highlighting some of the specific safety concerns they had prior to the School Street pilot:

*I was alarmed by the number of cars dropping off and picking up children from school during the 2020-2021 school year and worried that cars mingling with pedestrians, bikes, scooters, strollers had all the ingredients of a serious accident waiting to happen. When the opportunity arose of securing the street during peak student commute times, I was happy to volunteer.* (SSA AST advocate, parent, resident)

These worries, combined with the perceived potential for School Streets to provide an innovative solution to the traffic and safety problems, were highly motivating. Participants also described their interest and curiosity in the novel initiative as many were keen to know how a pilot project like this would work in their community. Participants from School Street B placed more emphasis on give back to their school community:

*I also love showing my daughter what active citizenship looks like, and community participation. This is healthy for her to see, that it's important to do even if we have ‘no time’ to [volunteer] as busy parents of young children.* (SSB parent)

Additionally, volunteers appreciated the physical and practical aspects of volunteer shifts, contrasting the regularity of this in-person volunteer position to the uncertainty brought on by the COVID-19 pandemic:

*Some of the biggest influences for me to start volunteering and STAY volunteering every week were the convenience of the morning hours (8:30-9:10), the minimal time commitment (40-80 minutes per week), and the proximity to [the university] (where I attended classes and have an office)...I enjoyed having the same shift every week and felt it “anchored” my week in the covid times of uncertainty, with classes moving on and offline.* (SSA university student)

Overall, community members were motivated to volunteer because they largely agreed with the School Street’s objectives, were drawn to the novelty of the initiative as a potential solution to safety concerns, and appreciated logistical aspects of the position, such as the minimal time commitment.

### Volunteers’ satisfaction

Table 3 illustrates the count and percentage of volunteers indicating ‘very satisfied’ feelings about different facets of their volunteer role. Overall, 91% of volunteers reported being very satisfied with the School Street initiative. In terms of volunteers’ perceptions regarding the outcomes of their work, 87% agreed that they increased the safety of children coming and going from school, 85% felt more integrated into the community since volunteering, and 72% agreed that they made new social connections through their involvement with the School Street (Table 4). Volunteers generally expressed a great deal of satisfaction, and even joy, with their volunteer experience:

*Experiencing the joy of coming around the corner… in the mornings and feeling the space that the School Streets project created to welcome students to school. This is in contrast to the dangerous and threatening feeling…when cars [were] parked on both sides of the street with traffic moving through the middle lane in both directions, while children are walking in all directions.* (SSA parent)

**Table 3:** Statistical Comparisons of Volunteers’ Satisfaction (*n* (%) reporting ‘very satisfied’) by School Street Site

Survey Question	Category	Pooled Total (*n*=56)	SS A (*n*=31)	SS B (*n*=25)	Chi-Square Statistic	Fisher's Exact Test (2-sided) *p*-value
		*n* (%)	*n* (%)	*n* (%)		
Overall, how satisfied were you with your experience volunteering for the SS program?	Overall satisfaction	50 (91)	27 (90)	23 (92)	0.066	1.000
How satisfied were you with the following specific aspects of this volunteer position?	Communications	53 (96)	29 (97)	24 (96)	0.017	1.000
	Shift length	53 (96)	30 (100)	23 (92)	2.491	0.202
	Scheduling	52 (95)	30 (100)	22 (88)	3.808	0.088
	Interactions with other volunteers	52 (95)	29 (97)	23 (92)	0.576	0.585
	Feeling appreciated by community organization	50 (93)	25 (86)	25 (100)	3.724	0.115
	Training	50 (91)	26 (87)	24 (96)	1.437	0.362
	Shift operations	41 (89)	28 (93)	21 (84)	1.222	0.394
	Shift timing	48 (89)	26 (90)	22 (88)	0.037	1.000
	Shift frequency	46 (85)	28 (97)	18 (72)	6.413	**0.019**
	Interactions with parents	45 (82)	23 (77)	22 (88)	1.177	0.318
	Feeling appreciated by parents	44 (82)	21 (72)	23 (92)	3.413	0.086
	Interactions with residents	28 (60)	8 (35)	20 (83)	11.495	**0.001**
	Feeling appreciated by school staff	28 (58)	9 (39)	19 (76)	6.7	**0.018**
	Feeling appreciated by residents	21 (49)	5 (28)	16 (64)	5.495	**0.031**

The *p*-values in bold represent differences between volunteers of School Street A and School Street B; bolded *p*-values are statistically significant at *p*<0.05

*SS* School Street

**Table 4:** Statistical Comparisons of Volunteers’ Agreement with Outcomes (*n* (%) reporting ‘strongly agree’) by School Street Site

Survey Question	Category	Pooled Total (*n*=56)	SS A (*n*=31)	SS B (*n*=25)	Chi-Square Statistic	Fisher's Exact Test (2-sided) *p*-value
		*n* (%)	*n* (%)	*n* (%)		
Indicate your level of agreement with the following outcomes related to your volunteer work for the SS.	I have made a meaningful contribution to the safety of children coming and going from the school	47 (87)	24 (83)	23 (92)	1.016	0.431
	I feel more like a part of the community since volunteering for the SS	46 (85)	24 (83)	22 (88)	0.292	0.711
	I made new social connections through my involvement with the SS	39 (72)	18 (62)	21 (84)	3.219	0.126

The *p*-values represent differences between volunteers of School Street A and School Street B

*SS* School Street

When asked to rate their satisfaction with specific elements of the initiative, volunteers were most satisfied with communications (96% very satisfied), shift length (96% very satisfied), scheduling (95% very satisfied), and interactions with other volunteers (95% very satisfied). Conversely, volunteers were least satisfied with feeling appreciated by residents (49% very satisfied) and school staff (58% very satisfied), though volunteers from School Street B experienced comparatively higher levels of satisfaction on both accounts (*p*= 0.031; *p*= 0.018, respectively). School Street B volunteers were also much more satisfied with their interactions with community residents compared to School Street A volunteers (*p*=0.001).

When asked to expand on any points that contributed to their level of satisfaction with the volunteer position, volunteers of School Street A remarked that the “*support of parents seemed high from the start”*, however they *“still sensed some tension with some residents and [school] staff”* (SSA parent, resident, AST advocate). Some elaborated, saying that there was resistance to the initiative by some residents during the first two months of the School Street’s operations, prior to a reduction in the length of the street closure:

*It was challenging to deal with some residents, especially during the first phase of the project when the scope was larger and there was a lot of resistance from certain neighbours; once the scope was narrowed down to eliminate those residents, things were much better.* (SSA AST advocate, resident)

Meanwhile, volunteers of School Street B did not comment on their interactions with residents or school staff. Despite some tension experienced by School Street A volunteers, they generally reported that the public’s appreciation for the initiative outweighed the minimal negative push-back: *“Some negative interactions with residents and school staff over the year, but mostly a positive experience!”* (SSA AST advocate, parent, resident).

Volunteers were satisfied with the community organization’s leadership: *“It was a lot of fun to hang out by the school and I was very impressed with the coordination”* (SSB parent, resident). One university student commented on the feasibility of fitting shifts into their schedule:

*Keeping shifts to 40 minutes (or less) in length makes it easy to fit into my day and influences my decision to continue volunteering, even during busy times such as the end of the semester.* (SSA university student)

Thus, volunteers strongly agreed that their contributions had improved the safety of children at the school and had made them feel more like a part of the community. There was also a high degree of satisfaction with many of the logistical (i.e., communications, shift length, scheduling) and social (i.e., interactions with parents and other volunteers, feeling appreciated by parents) aspects of the initiatives.

### Volunteer experiences

Beyond their satisfaction with the School Street, volunteers were also asked about the frequency with which they encountered issues during their shifts. Table 5 reveals that most issues were never or rarely experienced by volunteers at both sites, and showcases differences between volunteers of School Street A and School Street B through *p*-values. Inclement weather was the most frequently identified issue among both groups of volunteers (57% sometimes/often/always), followed by encountering aggressive or non-compliant motorists in the School Street zone for School Street A volunteers (46% sometimes/often/always). Despite some encounters with aggressive or non-compliant motorists, especially among School Street A volunteers, 96% of volunteers said they never/rarely experienced risks to their personal safety. Other issues that were rarely encountered by most volunteers included unauthorized motorists entering the School Street zone (84% never/rarely), difficulties accessing and/or setting up equipment (80% never/rarely), and other volunteers not showing up for shifts (77% never/rarely).

**Table 5:** Statistical Comparisons of Volunteers (*n* (%)) Reporting ‘Never or Rarely’ Encountering Issues by School Street Site

Survey Question	Category	Pooled Total (*n*=56)	SS A (*n*=31)	SS B (*n*=25)	Chi-Square Statistic	Fisher's Exact Test (2-sided) *p*-value
		*n* (%)	*n* (%)	*n* (%)		
How frequently did you encounter the following issues during your volunteer shifts?	Inclement weather	22 (43)	10 (36)	12 (52)	1.395	0.269
	Aggressive or non-compliant motorists within the SS zone	34 (67)	15 (54)	19 (83)	4.791	**0.039**
	Other volunteers not showing up for shifts	39 (77)	23 (82)	16 (70)	1.110	0.336
	Difficulties with accessing and/or setting up equipment	41 (80)	23 (82)	18 (78)	0.121	0.739
	Unauthorized motorists entering the SS zone	43 (84)	24 (86)	19 (83)	0.092	1.000
	Risks to your personal safety	49 (96)	27 (96)	22 (96)	0.020	1.000

The *p*-values represent differences between volunteers of School Street A and School Street B; bolded* p*-values are statistically significant at *p*<0.05

*SS* School Street

When given the chance to elaborate on issues encountered during their volunteer shifts, it was common for volunteers of School Street A to discuss “*unpleasant interactions with residents*” (SSA university student), such as instances with motorists “*insisting on entering the zone at the busiest time in the shift”* (SSA university student) or neighbours that “*made rude comments and did not follow the directions of the volunteers*” (SSA parent). Volunteers commented that negative interactions were infrequent and that community members were generally pleased and thankful for the support of volunteers within their community. Volunteers from both School Streets also commented on the types of issues they experienced related to equipment set-up and take down: *“The large metal sign is challenging to move and to break down, especially during the winter”* (SSB parent). An additional issue that emerged from the open-ended responses among School Street B volunteers was “*people trying to park in areas that were not parking spots - for example, blocking entryways to crosswalks*” (SSB parent).

Participants were also asked about their reasons for discontinuing their involvement in the School Street, and results are presented in Table 6. Across the two groups of volunteers, the most cited factor influencing their decision to stop was changes to their availability (86% very influential), while 27% of volunteers (most of whom were university students) had moved away from the area. A few volunteers were deterred by poor weather, logistical or social aspects of the position. When asked whether they would continue in the role if the School Street were to continue in the following year, 71% of the volunteers said ‘yes’. Volunteers were asked how they would like to be recognized for their time and effort, and most indicated their preference for a social event with other volunteers (34%), or that no recognition was necessary but a thank you card would be appreciated (26%). Less popular preferences included media attention, honoraria, and letters of reference.

**Table 6:** Statistical Comparison of Volunteers’ Reasons to Stop Volunteering (*n* (%) reporting ‘very influential’) by School Street Site

Survey Question	Category	Pooled Total (*n*=56)	SS A (*n*=31)	SS B (*n*=25)	Chi-Square Statistic	Fisher's Exact Test (2-sided) *p*-value
		*n* (%)	*n* (%)	*n* (%)		
How infuential were the following factors in your decision to stop volunteering for the SS initiative?	My availability changed	19 (86)	11 (79)	8 (100)	1.985	0.273
	Moved away from the area	6 (27)	4 (29)	2 (25)	0.033	1.000
	Poor weather	2 (9)	2 (14)	0 (0)	1.257	0.515
	Program was too far from my home	1 (5)	1 (7)	0 (0)	0.599	1.000
	Program was not what I expected	1 (5)	1 (7)	0 (0)	0.599	1.000
	Shifts were too long	0	0	0	Constant	Constant
	Shifts were too frequent	0	0	0	Constant	Constant
	Conflicts with motorists	0	0	0	Constant	Constant
	Felt unappreciated	0	0	0	Constant	Constant

The *p*-values represent differences between volunteers of School Street A and School Street B

*SS* School Street

Overall, the negative issues encountered by volunteers were generally infrequent and minor, stemming mostly from negative interactions with residents, and these issues did not appear to influence their decision to stop volunteering or their willingness to continue in the following year.

### Considerations for future programs

In two open-ended questions, volunteers were asked to provide their recommendations for improving the volunteer experience and for promoting the sustainability of School Streets. Volunteers suggested that the road closure equipment and equipment storage could be improved. One parent remarked on the need for heavy road closure signage:

*As a volunteer, a big part of the role was moving all of the [road closure] materials onto the street, some of which felt excessive. I understand this is what the city mandated, but in future years, it would be great if the degree of signage could be reduced. I’m mostly thinking of the very heavy stand for the road closure sign.* (SSB parent, resident)

Another volunteer suggested “*automatization of gates and signs*” as a potential solution (SSB resident). Moreover, a lack of dry and secure equipment storage space (e.g., a storage shed) on school property was a limitation to both of the School Streets’ operation. Volunteers were tasked with moving heavy equipment between various storage locations and the street. As equipment was stored outside year-round, *“winter conditions made accessing and setting up the equipment difficult at times”*, and obtaining dry and secure storage space would facilitate a timely set-up as well as reduce theft (SSA AST advocate, parent, resident). Secondly, volunteers felt it was important to improve the credibility of the School Street and wanted to see the municipal government or district school board take ownership of the initiative, and move away from the volunteer model:

*Having a direct and obvious connection to the city and school boards might alleviate some of the negative feelings we had to deal with and lend more credibility to the program.* (SSA AST advocate, resident)

*The need for volunteers should be temporary: Normally, you would expect the city, the school board, and the school working at permanent infrastructures that would ensure safety around schools.* (SSA parent)

Several volunteers extended this by saying that school staff or “*crossing guards could be trained to help, leading to only one volunteer needed per shift*” (SSB parent, resident). Volunteers also felt that the coordinator of the School Street should be paid:

*Coordination is a huge role, and I worry about our ability to sustain the program without a dedicated coordinator. I think this should be a paid position or incorporated into an existing paid position with the school, [community organization], or the city.* (SSB parent, resident)

## Discussion

### Key findings

The objective of this research was to understand the motivations and experiences of community volunteers whose collective labour enabled two School Streets to run every school day, twice a day, each for an entire academic year. Several key findings emerged from the surveys. Similar to other Ontario School Street pilots, the Kingston School Streets were run by a diverse pool of volunteers (consisting of parents, university students, and nearby residents) [23]. Overall, volunteers were highly committed (the majority delivering 1-2 shifts per week for more than 7 of the 10-month school year). Despite the group’s diversity, volunteers were commonly motivated by their concerns about the safety of the streets adjacent to the schools, and their excitement about reimagining how the street could be used.

Some interesting differences emerged between volunteers of School Street A and School Street B in terms of their motivations for volunteering. Volunteers of School Street A were more strongly motivated to promote AST and physical activity among children, whereas volunteers of School Street B were more strongly motivated to give back to their community and their child(ren)’s school. We suspect that the differences in these motivations between sites can be explained by a few reasons. The school where School Street A was implemented had relatively high rates of AST among the student body prior to the intervention being implemented, reflecting positive parental attitudes towards AST and daily physical activity within this neighbourhood. Indeed, the parents who volunteered for School Street A were those whose children used AST to get to and from school every day, and who felt the conditions could be improved for their children’s journeys. School Street A was also heavily supported by graduate students in urban planning and public health programs at the nearby university, which also explains the heavier emphasis on promoting AST and health in their motivations. In contrast, the neighbourhood surrounding School Street B is a highly socially cohesive neighbourhood with a long history of community-based activism [31]. Thus, the individuals who volunteered for School Street B were likely drawn to the disruptive nature of the intervention, and the opportunity to support their community’s well-being in a tangible way.

There was also a high degree of satisfaction among the volunteers with the outcomes of the initiatives (i.e., safety, community cohesion), as well as the logistical and social aspects of the initiative’s operations (e.g., communications, shift length, scheduling, and interactions with other volunteers). Problems while performing their volunteer shifts were infrequent, more commonly reported by volunteers of School Street A, and primarily limited to negative interactions with aggressive and non-compliant motorists. Finally, volunteers relayed critical considerations for future initiatives, including equipment storage, initiative ownership, and coordination.

Volunteers’ motivations and experiences of the School Street were critical to their recruitment and retention. Previous research has found a positive association between volunteering for reasons that are mainly driven by personal or altruistic values and volunteer commitment duration [32]. Findings from this study support this relationship; School Street volunteers’ motivations were largely rooted in personal values related to safety and health, and the majority were long-term volunteers. This research also aligns with previous literature showing that volunteers experience lower rates of burnout when their reasons for volunteering are satisfied by their volunteer experience [13]. School Street volunteers were particularly driven to promote safety around the school, and most also strongly agreed that they indeed had made a meaningful contribution to the safety of children. Volunteers were also satisfied with their intention to develop and strengthen social ties through this volunteer work. Most participants strongly agreed that they felt more like a part of the community since volunteering for the initiative and that they made new social connections through their involvement with the School Street. This is supported by previous findings identifying social connectedness as a strong predictor for duration of volunteer commitment [14].

While often overlooked, our study findings also emphasize the importance of logistical factors in volunteer retention. Indeed, previous studies have found that logistical aspects of the volunteer experience such as effective training, scheduling, consistent communication, and ongoing support are important factors for volunteer satisfaction and retention [11, 32–34]. In this study, most volunteers were highly satisfied with communications, which included weekly update emails, prompt responses, sharing positive feedback with the entire group, and involving volunteers in discussions regarding future changes. Volunteers were also highly satisfied with shift length (i.e., 40 minutes), and scheduling, which included estimating the number of volunteers required for each shift and distributing the upcoming week’s schedule consistently on Fridays. Moreover, the schedule offered regularity for those volunteers who needed regularity, and flexibility for those who needed flexibility; volunteers were consistently assigned to the same shift unless they encountered a scheduling conflict. School Street volunteers were also satisfied with training, which consisted of a 40-minute training session prior to volunteers’ first shift, and the distribution of an information sheet outlining the volunteer role and responsibilities.

Volunteers’ responsibilities for the School Streets initiatives were straightforward, and there was immediate positive reinforcement from the students and parents which made the experience positive overall. Maas et al. [34] similarly found that organizing projects with ‘a clear beginning and end’ and achieving visible results was important to volunteers’ sense of productivity, and accordingly their role satisfaction. Our findings suggest that enhancing the ease of barrier set-up among volunteers, or eliminating the need for it altogether, would enhance the overall volunteer experience. These findings contrast those of Mass et al [34], who found that physically demanding work promotes a sense of productivity and role satisfaction among volunteers of 1-day events. These contrasting findings could be explained by the nature of the volunteer work involved, and the assumption that volunteers of 1-day events may be less concerned with potential adverse effects of repetitive movements or heavy lifting compared to volunteers contributing 2 shifts per week for 7 months. Moreover, as some of the volunteers for the initiatives were retirees from the community, a greater portion of volunteers in this study may have experienced physical mobility constraints [23]. Overall, the literature on individual-level determinants of volunteering is far more extensive than the literature on determinants of contextual and physical factors such as volunteering conditions, equipment, and physical demands [35]. This research extends the existing literature, revealing the importance of both contextual and individual-level factors.

The volunteers of Kingston’s School Streets were strongly motivated to modify the environment in front of the school in ways that both protected against physical hazards and supported active living. The opportunity to reimagine how streets are used provided additional motivation for volunteers to disrupt the status quo of unsupportive (e.g., automobile-centric) environments. Although this generated resistance from stakeholders who benefit from the status quo, the ability to implement socially innovative initiatives without generating social tension is a common challenge [36–38]. Indeed, making physical changes to the streetscape to improve walkability was largely appreciated by parents and students in Kingston, however these changes were not appreciated by some residents who became resentful and hostile towards volunteers. Moreover, our findings that volunteers from School Street A felt less appreciated by school staff and residents, and experienced aggressive or non-compliant drivers more often than volunteers from School Street B suggests that the project scope and design are important considerations. For example, the number of dwellings, rate of vehicle ownership, and staff parking lot location differed between School Streets A and B, and these factors likely contributed to the varying levels of satisfaction among the two groups of volunteers in terms of feeling appreciated by school staff and residents. To minimize resistance to these innovative initiatives, implementers of future School Streets must consider the specific social and environmental contexts (e.g., staff parking lot location) when selecting a site and when determining the scope of street closures. Other implementers of School Street pilots have also noted the importance of scope, and that the design of each School Street should be determined based on the goals of the community [23].

By design, AST initiatives are expected to lead to increases in AST and higher self-efficacy for AST among school-aged children and their families [6, 39]. In the case of the Kingston School Streets, positive outcomes were also realized for the volunteers themselves and for the wider community, thereby strengthening community action for health promotion. Volunteers reported an increased quantity and quality of social connections in their community following their involvement. This could be, in part, due to the regularity of shifts (i.e., most volunteers took on one or two shifts per week), and positive interactions they experienced with other volunteers, parents, and children. The volunteer-led model also resulted in greater community cohesion. Kingston’s School Streets involved parents, grandparents, residents, university students, and other AST-advocates coming together to improve safety for children in the community. This diverse pool presented an opportunity for people to make connections around a common interest that they might not have otherwise made. The visibility and support of volunteers within the community sparked further engagement among community members. Consequently, School Street A was extended beyond the initial timeframe as volunteers were motivated to continue securing the street for the subsequent (2022-2023) school year.

### Implications for AST promotion

Volunteers were necessary for this pilot AST initiative and the pilot enabled municipal stakeholders to realize the transformative potential of such initiatives for creating health-supportive environments and community capacity building. These findings contribute to the AST literature by highlighting the characteristics of a distinct volunteer group whose efforts successfully sustained two year-long AST initiatives. The strengths and recommendations identified by the volunteers in this study are useful for other implementers of pilot AST initiatives who recruit volunteers to run their programs. Notably, recruiting a large and diverse group of volunteers (i.e., different life and career stages) is important for scheduling and sustaining these types of initiatives. Minimizing volunteer burden by keeping shifts short (i.e., 40 minutes), providing effective training, and ensuring that effort required to perform responsibilities is manageable for volunteers regardless of age and mobility limitations is important. The findings of this research also suggest that socially innovative health promotion initiatives have the potential to attract and retain volunteers through several key strategies. By clearly communicating the objectives and intended outcomes of the initiative, potential volunteers can understand the agency they have in contributing to health-promoting environments and the significance of their involvement. Emphasizing the innovative and disruptive aspects of School Streets and other street re-balancing initiatives may further motivate and encourage volunteers to remain involved.

Sustainability and equity remain key challenges in School Street implementation [40, 41]. While this community had the capacity to fulfil volunteer requirements, not all communities have the capacity to volunteer during the traditional workday. A few options for facilitating more sustainable School Streets include engineering approaches (e.g., installation of retractable bollards that elevate during designated School Street periods), enforcement approaches (e.g., issuing fines to unauthorized motorists who enter the closed zone through use of traffic cameras), or programmatic changes (e.g., expanding the school crossing guard model to have paid staff perform the same duties as School Street volunteers) [5, 41]. Regardless of the approach that is employed, collaboration and leadership from municipal governments and school boards is needed to develop sound strategies for ensuring the sustainability of School Streets specifically [41], and AST initiatives more broadly.

### Limitations and future research directions

Data for this project was drawn from volunteers who operated two School Street initiatives, each of which lasted for one academic year in Canada. Given the small sample size (*n*=56), our quantitative analyses were limited, and the questionnaire could not be piloted prior to administration. Development of a unique questionnaire allowed the researchers to tailor questions to this novel initiative and improve the questionnaire’s brevity. There were also limitations to the data generated by the open-ended responses, including the lower response rates to these questions and the inconsistency in the depth and applicability of responses given. This made it difficult for the research team to compare responses. Despite the challenges associated with including open-ended survey responses, the responses that were provided offered valuable contextual richness to the close-ended responses. Follow-up interviews with these volunteers would have added additional depth to our understanding of School Street volunteers’ motivations, satisfaction, and experiences with their role.

As more volunteer-run School Street initiatives from other municipal and sociodemographic contexts emerge, future research could compare the motivations, satisfaction, and experiences of these volunteer groups to the findings in this study. Such studies may reveal which factors are most important to sustaining these types of volunteer-run initiatives. Future research could also explore the extent to which volunteers’ participation in a School Street (or other AST initiative) shapes their willingness to become involved in other community-based initiatives.

## Conclusion

This paper presents a practical example of two communities that acted in two of the areas outlined by the Ottawa Charter, and the factors that compelled them to volunteer for a sustained period. This research suggests that socially innovative initiatives like School Streets may be more readily sustained by volunteers than other AST initiatives, as the volunteers in this study were highly motivated by the novelty and objectives of the School Street. In urban and suburban environments across Canada, School Streets offer promise for reshaping school travel behaviour and enhancing community cohesion, in light of their innovativeness. Our findings suggest that School Streets facilitate community building as individuals from diverse life and career stages came together voluntarily to prioritize and promote the health and safety of a younger generation and had satisfying social encounters during their volunteer shifts. Additionally, our research supports the idea that volunteer-led School Streets may be more readily accepted and sustained in neighbourhoods with unique social and economic features, specifically in those with a lower proportion of single-family dwellings, higher population density, and lower median household income. Future research should evaluate the impact of School Streets on community cohesion as well as modal shift in communities of contrasting socioeconomic status. Our findings also suggest that the scope of School Streets is important to their sustainability and the practical implications of these findings will be useful for implementers of future School Streets.

### Supplementary Information

**Supplementary Material 1.**

## Data Availability

The datasets generated and analysed during the current study are not publicly available to protect the privacy of participants but are available from the corresponding author on reasonable request.

## Footnotes

1 For the first 6 weeks, the scope of the street closure was 350 metres; it was later reduced to 192 metres in response to resident feedback and to reduce the overall number of volunteers required to operate the closure.
