# Supplementary material for: Promoting children’s health through community-led street interventions: analyzing sustained voluntarism in Canadian School Streets
Source: BMC Public Health. 2024 Apr 11;24:1011. doi: 10.1186/s12889-024-18531-9 (PMC11010341; doi:10.1186/s12889-024-18531-9)
Supplement: Supplementary file 1 — Supplementary Material 1. [file 12889_2024_18531_MOESM1_ESM.docx]

**Appendix A – Survey Instrument**

Q1 During which month did you start your work as a volunteer for the [name suppressed] School Street program?

▼ September (1) ... June (10)

Q2 During which month did you stop your work as a volunteer for the [name suppressed] School Street program?

▼ September (1) ... June/I am still volunteering (10)

Q3 During an average week, how many volunteer shifts did you work?

▼ 1 (1) ... 10 (10)

Q4 When did you typically do your shifts for the School Street program?

- Mornings (1)
- Afternoons (2)
- Both mornings and afternoons (3)

Q5 How influential were the following School Street program objectives in motivating you to volunteer for this program?

|  | Strongly influential (1) | Modestly influential (2) | Not influential (3) |
| --- | --- | --- | --- |
| Promoting active school travel (1) |  |  |  |
| Promoting children’s independent mobility (2) |  |  |  |
| Promoting safety around schools (3) |  |  |  |
| Promoting physical activity among children (4) |  |  |  |
| Re-imagining how streets can be used (5) |  |  |  |

Q6 How influential were these other factors in motivating you to volunteer for this program?

|  | Strongly influential (1) | Modestly influential (2) | Not influential (3) |
| --- | --- | --- | --- |
| Frequency of volunteer shifts (1) |  |  |  |
| Length of volunteer shifts (2) |  |  |  |
| Ease of work involved in a volunteer shift (3) |  |  |  |
| Proximity of the School Street to my home (4) |  |  |  |
| Opportunity to meet new people and socialize (5) |  |  |  |
| Opportunity to be outdoors (6) |  |  |  |
| Opportunity to be involved in a novel program (7) |  |  |  |
| Opportunity to be involved with community organization (8) |  |  |  |
| Opportunity to give back to my child(ren)’s school (9) |  |  |  |
| Opportunity to give back to my community (10) |  |  |  |
| Opportunity to gain volunteer experience for my career development (11) |  |  |  |

Q7 Please expand on the reasons why you decided to become involved as a volunteer for the School Street program.

________________________________________________________________

________________________________________________________________

Q8 Overall, how satisfied were you with your experience volunteering for the School Street program?

- Extremely satisfied (1)
- Very satisfied (2)
- Somewhat satisfied (3)
- Not very satisfied (4)
- Not at all satisfied (5)

Q9 How satisfied were you with the following specific aspects of this volunteer position?

|  | Extremely satisfied (1) | Very satisfied (2) | Somewhat satisfied (3) | Not very satisfied (6) | Not at all satisfied (7) |
| --- | --- | --- | --- | --- | --- |
| Training (1) |  |  |  |  |  |
| Communications (2) |  |  |  |  |  |
| Shift length (3) |  |  |  |  |  |
| Shift frequency (4) |  |  |  |  |  |
| Shift timing (5) |  |  |  |  |  |
| Shift operations (6) |  |  |  |  |  |
| Scheduling (7) |  |  |  |  |  |
| Interactions with other volunteers (8) |  |  |  |  |  |
| Interactions with parents (9) |  |  |  |  |  |
| Interactions with residents (10) |  |  |  |  |  |
| Feeling appreciated by parents (11) |  |  |  |  |  |
| Feeling appreciated by school staff (12) |  |  |  |  |  |
| Feeling appreciated by residents (13) |  |  |  |  |  |
| Feeling appreciated by community organization (14) |  |  |  |  |  |

Q10 Please expand on any points that contributed to your level of satisfaction with this volunteer position.

________________________________________________________________

________________________________________________________________

________________________________________________________________

Q11 Indicate your level of agreement with the following outcomes related to your work as a volunteer for the School Street program:

|  | Strongly Disagree (1) | Disagree (2) | Neutral (3) | Agree (4) | Strongly Agree (5) |
| --- | --- | --- | --- | --- | --- |
| I have made a meaningful contribution to the safety of children coming and going from the school (1) |  |  |  |  |  |
| I made new social connections through my involvement with the School Street (2) |  |  |  |  |  |
| I feel more like a part of the community since volunteering for the initiative (3) |  |  |  |  |  |

Display This Question:

If During which month did you stop your work as a volunteer for the [name suppressed] School Street... != June/I am still volunteering

Q12 How influential were the following factors in your decision to stop volunteering for the School Street program?

|  | Strongly influential (1) | Modestly influential (2) | Not influential (3) |
| --- | --- | --- | --- |
| My availability changed (1) |  |  |  |
| Moved away from the area (2) |  |  |  |
| Shifts were too long (3) |  |  |  |
| Shifts were too frequent (4) |  |  |  |
| Program was too far from my home (5) |  |  |  |
| Conflicts with motorists (6) |  |  |  |
| Program was not what I expected (7) |  |  |  |
| Poor weather (8) |  |  |  |
| Felt unappreciated (9) |  |  |  |

Display This Question:

If During which month did you stop your work as a volunteer for the Winston Churchill School Street... != June/I am still volunteering

Q13 Please expand on the reasons why you decided to stop volunteering for the School Street program.

________________________________________________________________

________________________________________________________________

________________________________________________________________

Q14 From your observations as a volunteer, to what extent do you think residents in the area approved of the program?

- Most disapproved (1)
- Some disapproved, some approved (2)
- Most approved (3)
- Don’t know (4)

Q15 What observation(s) led you to make this conclusion?

________________________________________________________________

________________________________________________________________

________________________________________________________________

Q16 How frequently did you encounter the following issues during your volunteer shifts?

|  | Never (1) | Rarely (2) | Sometimes (3) | Often (4) | Always (5) |
| --- | --- | --- | --- | --- | --- |
| Difficulties with accessing and/or setting up equipment (1) |  |  |  |  |  |
| Other volunteers not showing up for shifts (2) |  |  |  |  |  |
| Aggressive or non-compliant motorists within the School Street zone (3) |  |  |  |  |  |
| Unauthorized motorists entering the School Street zone (4) |  |  |  |  |  |
| Inclement weather (5) |  |  |  |  |  |
| Risks to your personal safety (6) |  |  |  |  |  |

Q17 Please expand on any issues that you encountered during your volunteer shifts.

________________________________________________________________

________________________________________________________________

________________________________________________________________

Q18 As a volunteer, how would you prefer to be recognized for your time and effort?

- Honorarium (1)
- Social event with other volunteers (2)
- Media attention (3)
- Letter of reference (4)
- Other, please specify: (5) __________________________________________________

Q19 If the School Street program ran next year at [name suppressed] School, would you consider volunteering?

- Yes (1)
- Maybe (2)
- No (3)

Q20 How could the volunteer experience be improved? Feel free to compare to other volunteer experiences, if applicable.

________________________________________________________________

________________________________________________________________

________________________________________________________________

Q21 How do you identify as a member of the community? Select all that apply.

- Parent of a [name suppressed] School student (1)
- Resident of the area (2)
- University student (3)
- Active school travel advocate (4)
- Other, please specify: (5) __________________________________________________

Q22 Please provide your postal code to enable us to understand how far volunteers were willing to travel for this opportunity.

________________________________________________________________

Q23 Based on your experience, do you have suggestions on how to promote the sustainability of School Streets for future years and/or at other sites?

________________________________________________________________

________________________________________________________________

________________________________________________________________
